# The prevalence of rheumatic diseases in central Greece: a population survey

**DOI:** 10.1186/1471-2474-11-98

**Published:** 2010-05-26

**Authors:** Ioannis Anagnostopoulos, Elias Zinzaras, Ioannis Alexiou, Aphrodite A Papathanasiou, Evangelos Davas, Athanasios Koutroumpas, Georgia Barouta, Lazaros I Sakkas

**Affiliations:** 1 Department of Rheumatology and Biomathematics, Thessaly University School of Medicine and Hospital, Larissa, 41 110, Greece

## Abstract

**Background:**

Rheumatic diseases are a major health and financial burden for societies. The prevalence of rheumatic diseases may change over time, and therefore, we sought to estimate the prevalence of rheumatic diseases in an adult population of central Greece.

**Methods:**

In this prospective cross-sectional population survey, a random sample of adult population was drawn from poll catalogues of a region in central Greece. A postal questionnaire was sent to 3,528 people for the presence of any rheumatic disease. All positive cases were further confirmed by clinical examination using the American College of Rheumatoloy criteria. Multiple regression analysis was used to assess risk factors for rheumatic diseases.

**Results:**

The response rate was 48.3% (1,705 answers). Four hundred and twenty individuals (24.6%) had a rheumatic disease. The prevalence of rheumatoid arthritis was 0.58% (95% confidence interval [CI], 0.32-0.87), of psoriatic arthritis was 0.35% (95% CI, 0.33-1.13), of ankylosing spondylitis was 0.29% (95% CI, 0.28-0.94), of primary Sjögren's syndrome was 0.23% (95% CI, 0.22-0.75) and of systemic lupus erythematosus was 0.11% (95% CI, 0.11-0.37). One individual had systemic sclerosis (prevalence, 0.058%), 1 individual had dermatomyositis (prevalence, 0.058%; 95% CI, 0.05-0.18), 2 individuals had vasculitis (prevalence 0.11%; 95% CI, 0.11-0.37), 81 individuals had gout (prevalence, 4.75%; 95% CI, 4.41-5.13), and 304 individuals had osteoarthritis (OA) (prevalence 17.82%; 95% CI, 16.50-19.34). Gout was associated with male gender, diabetes mellitus, and hypertension, and OA was associated with age, female gender, and hypertension.

**Conclusions:**

Rheumatic diseases are common in central Greece, affecting nearly a quarter of adult population. OA and gout are the most common joint disorders.

## Background

Rheumatic diseases are a common cause of disability, and a large public health burden [1]. However, they may vary considerably in prevalence, mainly due to environmental factors. For example, the prevalence of systemic lupus erythematosus (SLE) or systemic sclerosis (SSc) differ in ethnic populations living in different geographical regions [2,3]. Also, the prevalence of the rheumatic diseases may change over time. In a US population, the incidence of rheumatoid arthritis (RA) progressively declined since early 1960s, while the prevalence of gout doubled from 1969 to 1985, and it further increased by 80% from 1990 to 1999 [4]. In Greece, two older studies on the prevalence of rheumatic diseases have taken place mainly in the north-west of the country [5,6]. Therefore, we conducted a population survey for the prevalence of rheumatic diseases in central Greece.

## Methods

A prospective two-step population-based cross-sectional survey was conducted from April 2007 to June 2008 in the Prefecture of Magnesia, a coastal region of central Greece. This region consists of a mainland with plains, mountains, and islands. The study involved a mailed questionnaire followed by confirmation clinical examination and tests, when needed, of individuals with a positive reply.

A sample of adults was drawn from poll catalogues of the Prefecture of Magnesia by systematic random sampling. The study involved 3,528 subjects (2% of the total 176,433 adult population in poll catalogues), consisting of 2% of the population in each sub-region, urban, rural, highlander, and islander population. According to their sub-region of residence, our sample consisted of 2,216 persons living in urban regions (2% of urban adult population), 723 persons living in rural regions (2% of rural adult population), 335 persons living in mainland highlands (2% of adult highlanders), and 254 persons living in islands (2% of islander adult population). Persons who could not be traced by mail (n = 200) were replaced using the same randomization process.

A postal questionnaire aimed at detecting any individual with current or previous diagnosis of a rheumatic disease. An explanatory letter was sent along with the questionnaire in a stamped self-addressed envelope to each one of randomly selected individuals. Persons were asked directly to report any diagnosis of: rheumatoid arthritis (RA), psoriatic arthritis (PsA), ankylosing spondylitis (AS), systemic lupus erythematosus (SLE), primary Sjogren's syndrome (pSS), systemic sclerosis (SSc), polymyositis (PM), dermatomyositis (DM), gout, osteoarthritis (OA), and vasculitis (e.g Do you suffer from rheumatoid arthritis? yes or no; Do you suffer from psoriatic arthritis? yes or no; etc). These "self-reported doctor-diagnosed" questions seemed to provide the best estimate for overall arthritis prevalence, with acceptable sensitivity and specificity, according to cognitive and validation studies [4].

The questionnaire also contained questions about demographic and life-style variables. Social-demographic variables included age, sex, and working status (employed - current occupation, out of work [unemployed, early retirement] and retirement). A qualitative score (0 and 1) was given for the degree of mechanical stress to joints according the particular occupation (Table 1). Lifestyle variables included: smoking status, recorded as any current and/or in the past daily smoking (yes) or not (no); alcohol consumption, classified into 4 categories: no use (no more than 3 drinks per year), light drinking (less than 2 drinks per day), moderate drinking (2-4 drinks per day) and heavy drinking (more than 4 drinks per day). Financial and family problems were self-reported as yes/no variables. The utilization of classical disease modifying anti-rheumatic drugs (DMARDs) and biologics were also recorded.

**Table 1:** Demographic and lifestyle variables included in the questionnaire

Variable	Definition
Gender	Male/Female
Age	≥ 50 years
Residence	Urban, Rural, Highland, Island
Drinking situation	No use: ≤ 3 drinks per year Light drinker: < 2 drinks per day Moderate drinker: 2-4 drinks per day Heavy drinker: > 4 drinks per day
Current occupation	nonmanual: clerks, small business owners, merchants, university graduates, students, artists manual: Industry workers, construction workers, cleaning, farmers, ranchers, fishermen, transporters Out of work: unemployed, early retired Age retirement
Smoking status	Yes: Current and/or in the past daily smoking
	No: not ever daily smoking
Financial problems	Yes/No
Diabetes Mellitus	Yes/No
Hypertension	Yes/No

Case detection through questionnaire was followed by case confirmation. Individuals who responded positively for any disease item were called in for evaluation by one rheumatologist (IA). A medical history was recorded and a careful clinical examination was carried out. Any previous laboratory test results or imaging findings were taken into account during the diagnostic procedure. For each rheumatic disease, established classification criteria of the American College of Rheumatology were used [7-15]. The study was approved by the Ethics Committee of our Hospital. All data remained confidential.

### Statistical analysis

Statistical analysis was carried out using the SPSS v.13.0 statistics package. Categorical parameters were compared using the Fisher's exact test or Pearson chi-square test. Rheumatic diseases were tested for associations with gender, age, or other confounding parameters using univariate and multivariate logistic regression analysis. Associations were expressed in terms of unadjusted or adjusted odds ratio (OR) with 95% confidence interval (CI). An effect was considered significant, when p < 0.05.

## Results

One thousand seven hundred and five individuals (48.3%) (females, 54.1%; mean age ± standard deviation [SD], 51.08 ± 15.25 years) responded to questionnaire. Four hundred and twenty individuals (24.6%) reported a rheumatic disease and were invited for confirmation of diagnosis. Diagnoses had been made by Rheumatologists at University Hospitals, and private practice offices. All cases were confirmed.

Ten individuals had RA (7 women, 3 men; mean age ± SD, 46.82 ± 14.9 years; prevalence, 0.57% [95% CI, 0.32-0.87]). Eleven individuals had spondylartropathies (SpA): five individuals had AS (1 woman, 4 men; mean age ± SD, 43.46 ± 15.04 years; prevalence, 0.29% [95% CI, 0.28-0.94]), and six individuals had PsA (4 women, 2 men; mean age ± SD, 41.56 ± 2.2 years; prevalence, 0.35% [95% CI, 0.33-1.33]). Two individuals had SLE, (prevalence, 0.11%), four individuals had pSS (prevalence 0.23%; 95% CI, 0.22-0.75), one individuals had SSc (prevalence, 0.058%), and one individual had DM (prevalence 0.058%). Two individuals had vasculitis (prevalence, 0.11%): one with giant cell arteritis, and one with hypocomplementemic urticarial vasculitis syndrome (HUVS) (Table 2).

**Table 2:** Prevalence of rheumatic diseases in central Greece

Disease	Prevalence(%)	Age(years)	Women:men ratio
	(95% CI)	(mean ± SD)	
Rheumatoid arthritis	0.57 (0.32-0.87)	46.8 ± 14.9	2.3: 1
Ankylosing spondylitis	0.29 (0.28-0.94)	43.5 ± 15.0	1: 4
Psoriatic arthritis	0.35 (0.33-1.33)	41.6 ± 2.2	2:1
Sjogren syndrome	0.23 (0.22-0.75)		
Systemic lupus erythematosus	0.11		
Systemic sclerosis	0.06		
Dermatomyositis	0.06		
Vasculitis	0.11		
Gout	4.71 (4.41-5.13)	63.0 ± 7.8	0:8
Osteoarthritis (OA)	17.82 (16.5-19.34)	65.6 ± 7.7	2.7:1

Eighty one individuals had gout (mean age ± SD, 63.0 ± 7.8 years; prevalence, 4.71% [95% CI, 4.41-5.13]). The prevalence of gout was 10.0% in men and 0.3% in women. It was 0.0% in the < 40 year-age group, 5.3% in the 40-65 year-age group and 9.6% in the > 65 year-age group. By univariate logistic regression analysis, age, male gender, diabetes mellitus, hypertension, smoking, and manual work were risk factors associated with gout (Table 3).

**Table 3:** Variables assessed for association with gout

Variable		Gout		Unadjusted OR	P-value	Adjusted OR
		Yes	No	(95% CI)		**95% c.i**.
Gender	Male	10.0%	90.0%	33.926 (10.664-107.932)	<0.01	25.873 (7.811-85.699)
	Female	0.3%	99.7%			

Age	≥66	9.6%	90.4%	Non-applicable	<0.01	
	≥40 & <66	5.3%	94.7%			
	<40	0.0%	100.0%			

Residence	Island	6.2%	93.8%	1.446 (0.805-2.599)	0.346	1.107 (0.580-2.113)
	Mountain	6.1%	93.9%	1.016 (0.433-2.384)		0.738 (0.296-1.839)
	Urban and suburban	4.4%	95.6%	Reference		Reference

Alcohol	Yes	7.8%	92.2%	3. 357 (2.060-5.471)	<0.01	1.654 (0.819-3.342)
	No	2.5%	97.5%			

Smoke	Yes	7.4%	92.6%	2.589 (1.633-4.105)	<0.01	1.035 (0.540-1.984)
	No	3.0%	97.0%			

Financial problems	Yes	4.0%	96.0%	0.799 (0.490-1.305)	0.400	0.842 (0.489-1.450)
	No	5.0%	95.0%			

Manual work	Yes	6.5%	93.5%	1.680 (1.040-2.713)	0.044	0.973 (0.569-1.664)
	No	3.9%	96.1%			

Diabetes mellitus	Yes	11.1%	88.9%	2.972 (1.711-5.161)	<0.01	2.634 (1.350-5.138)
	No	4.0%	96.0%			

Hypertension	Yes	10.2%	89.8%	3.508 (2.218-5.547)	<0.01	2.780 (1.649-4.686)
	No	3.1%	96.9%			

Three hundred and four individuals had OA (mean age ± SD, 65.54 ± 12.9 years; prevalence, 17.82 [95% CI, 16.5-19.34]). The overall prevalence in women was 12.96% (95% CI, 11.85-14.23; mean age ± SD, 72.39 ± 8.25 years), and in men 4.86% (95% CI, 4.35-5.43; mean age ± SD, 65.97 ± 7.57 years). The prevalence of symptomatic knee OA (knee symptoms verified by a positive radiograph) was 11.3% (95% CI, 9.76-13.09) in women, and 2.99% (95% CI, 2.6-3.42) in men. Also, the prevalence of hand OA was 0.99% (95% CI, 0.76-1.25) in women, and 0.70% (95% CI, 0.56-0.86) in men. Finally, the prevalence of symptomatic hip OA was 0.64% (95% CI, 0.43-0.89) in women, and 1.17% (95% CI, 0.99-1.38) in men. By univariate logistic regression analysis, age, diabetes mellitus, hypertension, and living in island were risk factors associated with OA (Table 4).

**Table 4:** Variables assessed for association with osteoarthritis

Variable		Osteoarthritis		Unadjusted OR	P-value	Adjusted OR
		Yes	No	(95% CI)		**95% c.i**.
Gender	Male	11.3%	88.7%	0.411 (0.314-0.538)	<0.01	0.430 (0.306-0.604)
	Female	23.6%	76.4%			
Age	≥66	39.7%	60.3%	265.240 (36.807-1911.358)	<0.01	173.679 (23.714-1271.983)
	≥40 & <66	18.4%	81.6%	2.926 (2.220-3.857)		2.140 (1.541-2.974)
	<40	0.2%	99.8%	Reference		Reference
Residence	Island	23.8%	76.2%	1.546 (1.113-2.148)	0.033	1.186 (0.810-1.737)
	Mountain	18.2%	81.8%	1.397 (0.838-2.329)		1.656 (0.919-2.983)
	Urban & suburban	16.8%	83.2%	Reference		Reference
Alcohol	Yes	9.9%	90.1%	0.352 (0.264-0.468)	<0.01	0.827 (0.534-1.283)
	No	23.7%	76.3%			
Smoke	Yes	9.2%	90.8%	0.334 (0.247-0.451)	<0.01	0.667 (0.425-1.045)
	No	23.3%	76.7%			
Financial problems	Yes	20.8%	79.2%	1.353 (1.049-1.745)	0.020	1.316 (0.984-1.759)
	No	16.3%	83.7%			
Manual work	Yes	13.8%	86.2%	0.649 (0.477-0.882)	0.006	1.044 (0.727-1.499)
	No	19.8%	80.2%			
Diabetes	Yes	29.0%	71.0%	2.045 (1.420-2.946)	<0.01	0.739 (0.488-1.117)
	No	16.7%	83.3%			
Hypertension	Yes	36.7%	63.3%	3.987 (3.051-5.210)	<0.01	2.131 (1.553-2.926)
	No	12.7%	87.3%			

## Discussion

Our study is a population-based postal survey using a random population sample. In Manchester, UK, a study by questionnaire followed by examination conducted in early 1990s, showed that the prevalence of RA was 0.3% in blacks and 0.8% in whites [16]. In Halmstad, Sweden, a study using a questionnaire of 3,928 persons and review of medical records of persons who did not reply, found a prevalence of RA to be 0.51% [17]. In Spain and Italy, the prevalence of RA was 0.3-0.5% [18,19]. In our study, the prevalence of RA lies between those found by a retrospective study (0.35%) [5] and a cross-sectional study (0.68%) [20] in Greece.

The prevalence of AS varies according to the frequency of HLA-B27 in the general population. It has been reported 0.1% in African blacks and Eskimos, 0.5%-1% in whites of the USA and UK, and 6.0% in Amerindians [21]. In a retrospective study, using medical records of a University hospital in northern Norway in 1990, the prevalence of AS was 0.21% [22]. In Ancona, Italy, a study, using a questionnaire followed by review of medical records and examination in 2007, showed a prevalence of AS of 0.37%, and that of SpA of 1.1% [23]. In north-west Greece a study, using medical records of hospitals and Rheumatology Private Practices between 1983 and 2002, has found AS prevalence to be 0.03%, with HLA-B27 positivity present in 80.5% of patients [24]. Another study in Greece in 2002 showed a prevalence of AS of 0.24% and that of SpA of 0.49% [6]. In Izmir, Turkey, the prevalence of AS was 0.49% and that of SpA was 1.05% [25].

The prevalence of PsA in a study based on medical records in the Olmsted county, Minesota, USA in 1992 was 0.1% [4,26] and in another study conducted by phone in a random US population sample was 0.25% [27]. In the latter study, the prevalence of psoriasis was 2.2% [27]. In western Norway, a study using medical records of Rheumatology centers covering 442,000 persons between 1999 and 2002, found the prevalence of AS to be 0.19% [28]. In Ancona, Italy, a study using questionnaire in 2007 showed a prevalence of PsA of 0.42% [23]. Our PsA prevalence estimate is similar to that reported earlier in Greece (0.17%) [6].

Our estimate of SLE prevalence (0.11%) is slightly higher than that reported in a study using medical records of Hospitals and rheumatology practices in northwest Greece in 2001 (0.009% for men, 0.069% for women) [29]. The prevalence of SLE was 0.05% in a study using physician billing and hospital database in Quebec, Canada [30], and 0.01% in males and 0.071% in females in a study using General Practice Research Database in UK [31]. In northern Spain, a study using database of the single Immunology laboratory of a region, found SLE prevalence to be 0.034% [32], and in Northern Italy, a study using hospital medical records reported SLE prevalence 0.058% [33].

The prevalence of pSS in our study (0.23%) is in agreement with those of two previous community-based studies in Greece [34,35]. In Norway, the prevalence of pSS, as detected by postal questionnaire, was 0.22% in adult ages 40-44 years and 1.4% in the adult ages 71-74 years [36], and in UK the prevalence of pSS was 0.33% [37].

Our finding that only one person was diagnosed with SSc, DM, giant cell arteritis, hypocoplementemic urticarial vasculitis precludes any comment on the prevalence of these diseases. However, it is worth mentioning that there are geographical clusters of SSc which suggest an environmental aetiology for the disease [3].

The prevalence of gout increases with age, is higher in men than women, and is on the increase. In adult age >75 years in USA, the prevalence of gout increased from 2.1% in 1990, to 4.1% in 1999 [38]. The respective values for men were 2.5%, and 6.4% [38]. In the US National Health and Nutrition Examination Survey (NHANES) III between 1988 and 1994, the highest prevalence (8%; males, 11.6%; females, 5.2%) was found in adult ages 70-79 years [39,40]. In a UK study using General Practitioner database, the prevalence of gout was 0.3% in the 1970s, and 0.95% in the 1990s [41]. Results from the UK General Practice database during the period 1990-1999 showed relatively constant incidence rates. In 1999 the prevalence of gout was 1.4% in adult population, and 7.0% in males over 65 years of age [42]. A retrospective study using medical records of General Practitioners in UK and Germany between 2000 and 2005 found the prevalence of gout to be 1.4% (mean age, 60 years) [43]. In China, the prevalence of hyperuricemia and gout varied among different regions. The prevalence of gout was 11.7% in Taiwanese aborigines and 3.0% in ethnic Taiwanese [44]. Our estimate of the prevalence of gout is much higher than that reported in the general Greek population (0.47%) a few years ago [6]. This may be explained by the relatively old age of our gouty patients (mean age, 63 years), and the changing diet and lifestyle. High seafood consumption, which is a risk factor for gout [45], is prevalent in this coastal region of Greece. Our study has found gout to be associated with male gender, hypertension, and diabetes mellitus. Since hypertension and type II diabetes mellitus are components of the metabolic syndrome or insulin resistance syndrome, our gouty men are likely to have this syndrome that carries an increased cardiovascular risk [46].

Epidemiological studies of OA have inherent problems due to lack of association between symptoms and radiographic findings that may underestimate OA prevalence. Generally, the prevalence of OA is associated with age and is higher in women. The prevalence of symptomatic hand OA, verified by x-rays, in the Framingham study was 6.8% (3.8% in men, 9.2% in women) whereas in adults age > 71 years was 13.4% in men and 26.2% in women [47]. The prevalence of symptomatic knee OA was 4.9%, and increased to 6.7% (5.9% in men, 7.2% in women) in adults aged > 45 years [48]. In the US NHANES III study, the prevalence of symptomatic knee OA in adults age >60 years was 12.1% [49], and in the Jonston county, northern Carolina, USA, it was 16.7% in adults age > 45 years [50]. In the latter study, the prevalence of symptomatic hip OA was 9.2% [40]. In British Columbia, Canada, database from Health centers and Hospitals between 1991 and 2001 showed a prevalence of symptomatic OA in 2001 of 10.0% in adult ages 45-49 years, whereas 32% of men and 40% of women aged 70 years had OA [51]. In France, a phone survey in a random population sample of adults aged 40-75 years found the prevalence of knee OA and hip OA to be 7.6% and 5%, respectively [52]. In Spain, the frequency of symptomatic hand OA was 6.2% and knee OA 10.2% [53]. In our study, there is a low frequency of hand OA. Individuals with hand OA may have mild symptoms and do not seek medical advice. Therefore, these patients did not report their condition. The relatively low prevalence of OA in manual workers, compared to non-manual workers, may be attributed to women as house-wives. These women, included in non-manual workers, may have not only house duties but they may also work manually in the fields, a common practice in the country. A previous study in Greece showed a prevalence of symptomatic hand OA of 2.0%, knee OA 6.0%, and hip OA of 0.9% [54].

Radiographic OA, defined by the presence of osteophytes is much prevalent than symptomatic OA. In the Framingham study, the prevalence of radiographic hand OA was 27.2% (25.9% in men, 28.2% in women) [47]. The prevalence of radiographic knee OA was 13.8%, and increased to 19.2% in the > 45 year-old age group [48]. In the Jonston county study, the prevalence of radiographic OA in the >45 year age group was 27.8% (24.3% in men, 30.15% in women) and hip OA was 27.8% [40,50]. In the US NHANES III study, the prevalence of radiographic knee OA was 37.4% (31.2% in men, 42.1% in women) in the > 60 year-old age group [49]. In Holland, radiographic hand OA was found in 67% of women and 54.8% of men > 55 years of age [55].

As with other studies, our study has limitations to consider. Firstly, our study relied on patient self-reporting of a physician diagnosis of a disease as opposed to ascertaining a diagnosis through medical records. Additionally, our study may have missed patients with mild arthritis, such as OA, that did not come to medical attention. However, our estimates are generally close to those studies that used medical records to ascertain rheumatic disease diagnoses. Furthermore, self-report of chronic disease has been found accurate [56]. Secondly, our study had a relatively low response rate. However, similar response rate has also been reported by other postal surveys [57] and may be attributed to the increasingly use of questionnaires (postal or by phone) in public marketing.

## Conclusions

Rheumatic diseases are common in central Greece, affecting nearly a quarter of adult population. OA and gout are the most common joint disorders.

## Abbreviations

Abbreviations are defined in the text

## Competing interests

The authors declare that they have no competing interests.

## Authors' contributions

IA collected data, examined the patients, verified the diagnosis. EZ conceived the study design and performed the statistical analysis. IA verified the diagnosis. AP performed the statistical analysis. ED carried out literature search. AK wrote the manuscript. GB carried out literature search. LS conceived the study design and wrote the manuscript. All authors read and approved the final manuscript.

## Pre-publication history

The pre-publication history for this paper can be accessed here:

http://www.biomedcentral.com/1471-2474/11/98/prepub

## References

[B1] Yelin E Cisternas M Pasta D Trupin L Murphy L Helmick CG Medical care expenditures and earning losses of persons with arthritis and other rheumatic conditions in the United States in 1997:total and incremental estimates Arthritis Rheum 2004 50 2317 26 10.1002/art.20298 15248233

[B2] Fessel WJ Epidemiology of systemic lupus erythematosus Rheum Dis Clin North Am 1988 14 15 23 3041487

[B3] Chifflot H Fautrel B Sordet C Chatelus E Sibilia J Incidence and prevalence of systemic sclerosis: a systematic literature review Sem Arthritis Rheum 2008 37 223 35 10.1016/j.semarthrit.2007.05.003 17692364

[B4] Helmick CG Felson DT Lawrence RC Gabriel S Hirsch R Kent Kwoh C Liang MH Kremers HM Mayes MD Merkel PA Pillemer SR Reveille JD Stone JH Estimates of the prevalence of arthritis and other rheumatic conditions in the United States. Part I Arthritis Rheum 2008 58 15 25 10.1002/art.23177 18163481

[B5] Drosos AA Alamanos I Voulgari PV Psychos DN Katsaraki A Papadopoulos I Dimou G Siozos C Epidemiology of adult rheumatoid arthritis in northwest Greece 1987-1995 J Rheumatol 1997 24 2129 33 9375871

[B6] Andrianakos A Trontzas P Christogiannis F Dantis P Voudouris C Georgountzos A Kaziolas G Vafiadou E Pantelidou K Karamitsos D Kontelis L Krachtis P Nikolia Z Kaskani E Tavaniotou E Antoniades C Karanikolas G Kontoyanni A Prevalence of rheumatic diseases in Greece: a cross-sectional population based epidemiological study. The ESORDIG Study J Rheumatol 2003 30 1589 601 12858464

[B7] Wallace SL Robinson H Masi ET Decker JL Mc Carty DJ Yu TF Preliminary criteria for the classification of the acute arthritis of primary gout Arthritis Rheum 1977 20 895 900 10.1002/art.1780200320 856219

[B8] Bird HA Esselinckx W Dixon AS Mowat AG Wood PH An evaluation of criteria for polymyalgia rheumatica Ann Rheum Dis 1979 38 434 9 10.1136/ard.38.5.434 518143 PMC1000388

[B9] Subcommittee for Scleroderma Criteria of the American Rheumatism Association Diagnostic and Therapeutic Criteria Committee Preliminary criteria for the classification of systemic sclerosis (scleroderma) Arthritis Rheum 1980 23 581 90 10.1002/art.1780230510 7378088

[B10] Tan EM Cohen AS Fries JF Masi AT McShane DJ Rothfield NF Schaller JG Talal N Winchester RJ The 1982 revised criteria for the classification of systemic lupus erythematosus Arthritis Rheum 1982 25 1271 7 10.1002/art.1780251101 7138600

[B11] Arnett FC Edworthy SM Bloch DA McShane DJ Fries JF Cooper NS Healey LA Kaplan SR Liang MH Luthra HS The American Rheumatism Association 1987 revised criteria for the classification of rheumatoid arthritis Arthritis Rheum 1998 31 315 24 10.1002/art.1780310302 3358796

[B12] Hunder GG Bloch DA Mitchell BA Stevens MB Arend WP Calabrese LH Edworthy SM Fauci AS Leavitt RY Lie JT The American College of Rheumatology1990 criteria for the classification of giant cell arteritis Arthritis Rheum 1990 33 1122 8 2202311 10.1002/art.1780330810

[B13] Dougados M Linden S van der Juhlin R Huitfeldt B Amor B Calin A Cats A Dijkmans B Olivieri I Pasero G The European Spondyloarthropathy Study Group preliminary criteria for spondyloarthropathy Arthritis Rheum 1991 34 1218 27 10.1002/art.1780341003 1930310

[B14] Vitali C Bombardieri S Jonsson R Moutsopoulos HM Alexander EL Carsons SE Daniels TE Fox PC Fox RI Kassan SS Pillemer SR Talal N Weisman MH Classification criteria for Sjogren's syndrome: a revised version of the European criteria proposed by the American-European consensus group Ann Rheum Dis 2002 61 554 58 10.1136/ard.61.6.554 12006334 PMC1754137

[B15] Taylor W Gladman D Helliwell P Marchesoni A Mease P Mielants H for the CASPAR study group Classification criteria for psoriatic arthritis: development of new criteria from a large international study Arthritis Rheum 2006 54 2665 73 10.1002/art.21972 16871531

[B16] MacGregor AJ Riste LK Hazes JMW Silman AJ Low prevalence of rheumatoid arthritis in black-Caribbeans compared with whites in inner city Manchester Ann Rheum Dis 1994 53 293 7 10.1136/ard.53.5.293 8017981 PMC1005326

[B17] Simonsson M Bergman S Jacobsson LTH Petersson IF Svensson B The prevalence of rheumatoid arthritis in Sweden Scand J Rheumatol 1999 28 340 3 10.1080/03009749950155319 10665738

[B18] Cimmino MA Parisi M Moggiana G Mela GS Accardo S Prevalence of rheumatoid arthritis in Italy: the Chiavari study Ann Rheum Dis 1998 57 315 8 10.1136/ard.57.5.315 9741317 PMC1752594

[B19] Carmona L Villaverde V Hernandez-Garcia C Ballina J Gabriel R Laffon A for the EPISER study group The prevalence of rheumatoid arthritis in the general population of Spain Rheumatology 2002 41 88 95 10.1093/rheumatology/41.1.88 11792885

[B20] Andrianakos A Trontzas P Christoyiannis F Kaskani E Nikolia Z Tavaniotou E Georgountzos A Krachtis P for the ESORDIG study group Prevalence and management of rheumatoid arthritis in the general population of Greece-the ESORDIG study Rheumatology 2006 45 1549 54 10.1093/rheumatology/kel140 16690763

[B21] Baum J Ziff M The rarity of ankylosing spondylitis in the black race Arthritis Rheum 1971 14 12 8 10.1002/art.1780140103 5542364

[B22] Backland G Nossent HC Gran JT Incidence and prevalence of ankylosing spondylitis in Northern Norway Arthritis Rheum 2005 53 850 5 10.1002/art.21577 16342091

[B23] de Angelis R Salalfi F Grassi W Prevalence of spondyloarthropathies in an Italian population sample: a regional community-based study Scand J Rheumatol 2007 36 14 21 10.1080/03009740600904243 17454930

[B24] Alamanos Y Papadopoulos NG Voulgari PV Karakatsanis A Siozos C Drosos AA Epidemiology of ankylosing spondylitis in northwest Greece, 1983-2002 Rheumatology 2004 43 615 8 10.1093/rheumatology/keh133 14872102

[B25] Onen F Akar S Birlik M Sari I Khan MA Gourler O Ergor A Manisali M Akkoc N Prevalence of ankylosing spondylitis and related spondyloarthritides in an urban area of Izmir, Turkey J Rheumatol 2008 35 305 9 18085733

[B26] Shbeeb M Uramoto KM Gibson LE O' Fallon WM Gabriel SE The epidemiology of psoriatic arthritis in Olmsted County, Minesota, USA, 1982 - 1991 J Rheumatol 2000 27 1247 50 10813295

[B27] Gelfand JM Gladman DD Mease PJ Smith N Margolis DJ Nijsten T Stern RS Feldman SR Rolstad T Epidemiology of psoriatic arthritis in the population of the United States J Am Acad Dermatol 2005 53 573 77 10.1016/j.jaad.2005.03.046 16198775

[B28] Madland TM Apalset EM Johannessen AE Rossebo B Brun JG Prevalence, disease manifestations, and treatment of psoriatic arthritis in Western Norway J Rheumatol 2005 32 1918 22 16206347

[B29] Alamanos Y Voulgari PV Siozos C Katsimpri P Tsintzos S Dimou G Politi EN Rapti A Laina G Drosos AA Epidemiology of systemic lupus erythematosus in northwest Greece 1982-2001 J Rheumatol 2003 30 731 735 12672191

[B30] Bernatsky S Joseph L Pineau CA Tamblyn R Feldman DE Clarke AE A population-based assessment of systemic lupus erythematosus incidence and prevalence results and implications of using administrative data for epidemiological studies Rheumatology 2007 46 1814 1818 10.1093/rheumatology/kem233 18032538

[B31] Nightngale AL Farmer RD de Vries CS Systemic lupus erythematosus prevalence in UK: methodological issues when using the General Practice Research Database to estimate frequency of chronic relapsing-remitting disease Pharmacoepidimiol Drug Saf 2007 16 144 51 10.1002/pds.1253 16700093

[B32] Lopez P Mozo L Gutierrez C Suarez A Epidemiology of systemic lupus erythematosus in a northern Spanish population: gender and age influence on immunological features Lupus 2003 12 860 865 10.1191/0961203303lu469xx 14667105

[B33] Govoni M Gastellino G Bosi S Napoli N Trotta F Incidence and prevalence of systemic lupus erythematosus in a district of north Italy Lupus 2006 15 110 113 10.1191/0961203306lu2235xx 16539284

[B34] Dafni UG Tzioufas AG Staikos P Skopouli FN Moutsopoulos HM Prevalence of Sjogren's syndrome in a closed rural community Ann Rheum Dis 1997 56 521 525 10.1136/ard.56.9.521 9370875 PMC1752451

[B35] Trontzas PI Andrianakos AA Sjogren's syndrome: a population based study of prevalence in Greece. The ESORDIG study Ann Rheum Dis 2005 64 1240 41 10.1136/ard.2004.031021 16014690 PMC1755610

[B36] Haugen AJ Peen E Hulten B Johannessen AC Brun JG Halse AK Haga HJ Estimation of the prevalence of primary Sjogren's syndrome in two age-different community-based populations using the two sets of classification criteria: the Hordaland Health Study Scand J Rheumatol 2008 37 30 4 10.1080/03009740701678712 18189192

[B37] Thomas E Hay EM Hajeer A Silman AJ Sjogren's syndrome: a community based study of prevalence and impact Br J Rheumatol 1998 37 1069 76 10.1093/rheumatology/37.10.1069 9825745

[B38] Wallace KL Riedel AA Joseph-Ridge N Wortmann R Increasing prevalence of gout and hyperuricemia over 10 years among older adults in a managed care population J Rheumatol 2004 31 1582 7 15290739

[B39] Kramer HM Curhan G The association between gout and nephrolithiasis: the National health and Nutrition Examination Survey III, 1988-1994 Am J Kidney Dis 2002 40 37 42 10.1053/ajkd.2002.33911 12087559

[B40] Lawrence RC Felson DT Helmick CG Arnold LM Choi H Deyo RA Gabriel S Hirsch R Hochberg MC Hunder GG Jordan JM Katz JN Kremers HM Wolfe F Estimates of the prevalence of arthritis and other rheumatic conditions in the United States: Part II Arthritis Rheum 2008 58 26 35 10.1002/art.23176 18163497 PMC3266664

[B41] Harris CM Lloyd DCEF Lewis J The prevalence and prophylaxis of gout in England J Clin Epidemiol 1995 48 1153 58 10.1016/0895-4356(94)00244-K 7636517

[B42] Mikuls TR Farrar JT Bilker WB Fernandes S Schumacher HR Jr Saag KG Gout epidemiology: results from the UK general practice research database, 1990-1999 Ann Rheum Dis 2005 64 267 272 10.1136/ard.2004.024091 15647434 PMC1755343

[B43] Annemans L Spaepen E Gaskin M Bonnemaire M Malier V Gilbert T Nuki G Gout in the UK and Germany: prevalence, co-morbidities and management in general practice 2000-2005 Ann Rheum Dis 2008 67 960 6 10.1136/ard.2007.076232 17981913 PMC2564789

[B44] Nan H Qiao Q Dong Y Gao W Tang B Qian R Tuomilehto J The prevalence of hyperuricemia in a population of the coastal city of Qingdao, China J Rheumatol 2006 33 1346 50 16821269

[B45] Saag KG Choi H Epidemiology, risk factors, and lifestyle modification for gout Arthritis Res Ther 2006 8 Suppl 1 S2 7 10.1186/ar1907 16820041 PMC3226107

[B46] Puig JG Martinez MA Hyperuricemia, gout and the metabolic syndrome Curr Opin Rheumatol 2008 20 187 91 10.1097/BOR.0b013e3282f4b1ed 18349749

[B47] Zhang Y Niu J Kelly-Hayes M Chaisson CE Aliabadi P Felson DT Prevalence of symptomatic hand osteoarthritis and its impact on functional status among the elderly. The Framingham study Am J Epidemiol 2002 156 1021 7 10.1093/aje/kwf141 12446258

[B48] Felson DT Naimatk A Anderson J Kazis L Castelli W Meenan RF The prevalence of knee osteoarthritis in the elderly: the Framingham osteoarthritis study Arthritis Rheum 1987 30 914 18 10.1002/art.1780300811 3632732

[B49] Dilon CF Rasch EK Gu Q Hirsch R Prevalence of knee osteoarthritis in the United States: arthritis data from the third National Health and Nutrition Examination Survey 1991-94 J Rheumatol 2006 33 2271 79 17013996

[B50] Jordan JM Helmick CG Renner JR Luta G Dragomir AD Woodard J Fang F Schwartz TA Abbate LM Callahan LF Kalsbeek WD Hochberg MC Prevalence of knee symptoms and radiographic and symptomatic knee osteoarthritis in African Americans and Caucasians; the Johnston county osteoarthritis project J Rheumatol 2007 34 172 80 17216685

[B51] Kopec JA Rahman MM Berthelot JM Le Petit C Aghajanian J Sayre EE Cibere J Anis AH Badley EM Descriptive epidemiology of osteoarthritis in British Columbia, Canada J Rheumatol 2007 34 386 93 17183616

[B52] Roux CH Saraux A Mazieres B Pouchot J Morvan J Fautrel B Testa J Fardellone P Rat AC Coste J Guillemin F Euller-Ziegler L Screening for hip and knee osteoarthritis in the general population: predictive value of a questionnaire and prevalence estimates Ann Rheum Dis 2008 67 1406 11 10.1136/ard.2007.075952 18077540

[B53] Carmona L Ballina J Gabriel R Laffon A for the EPISER study group The burden of musculoskeletal diseases in the general population of Spain: results from a national survey Ann Rheum Dis 2001 60 1040 45 10.1136/ard.60.11.1040 11602475 PMC1753418

[B54] Andrianakos AA Kontelis LK Karamitsos DE Aslanidis SI Georgountzos AI Kaziolas GO Pantelidou KV Vafiadou EV Dantis PC for the ESORDIG study group Prevalence of symptomatic knee, hand, and hip osteoarthritis in Greece. The ESORDIG study J Rheumatol 2006 33 2507 13 17143985

[B55] Dalaghin S Bierma-Zeinstra SMA Ginai AZ Pols HAP Hazes JMW Koes BW Prevalence and pattern of radiographic hand osteoarthritis and association with pain and disability (the Rotterdam study) Ann Rheum Dis 2005 64 682 87 10.1136/ard.2004.023564 15374852 PMC1755481

[B56] Martin LM Leff M Calogne N Garrett C Nelson DE Validation of self-report chronic conditions and health services in a managed care population Am J Prev Med 2000 18 215 18 10.1016/S0749-3797(99)00158-0 10722987

[B57] Grotle M Hagen KB Natvig FA Dahl FA Kvien TK The prevalence and burden of osteoarthritis: results from a population survey in Norway J Rheumatol 2008 35 677 84 18278832

